# Cells Expressing Prominin-1 in Neonatal Murine Inferior Colliculus Differentiate into Neurons and Glia

**DOI:** 10.1007/s12035-017-0701-5

**Published:** 2017-08-09

**Authors:** Haruka Okazaki, Akira Kanda, Seiji Kanda, Takaki Shimono, Yasutaka Yun, Yoshiki Kobayashi, Zeyun Wang, Hisashi Ooka, Kensuke Suzuki, Dan Bui Van, Koichi Tomoda, Hiroshi Iwai, Toshimasa Nishiyama

**Affiliations:** 1 Department of Otolaryngology, Kansai Medical University|, Osaka, Japan; 2 Regeneration Research Center for Intractable Diseases, Kansai Medical University, Osaka, Japan; 3 Department of Public Health, Kansai Medical University, Osaka, Japan

**Keywords:** Inferior colliculus, Prominin-1, Neural stem cell, Central auditory pathway

## Abstract

Inferior colliculus (IC) is a major center for the integration and processing of acoustic information from ascending auditory pathways. Damage to the IC as well as normal aging can impair auditory function. Novel strategies such as stem cell (SC)-based regenerative therapy are required for functional recovery because mature neural cells have a minimal regenerative capacity after an injury. However, it is not known if there are neural stem cells (NSCs) in the IC. Herein, we screened for NSCs by surface marker analysis using flow cytometry. Isolated IC cells expressing prominin-1 (CD133) exhibited the cardinal NSC properties self-renewal capacity, expression of known NSC markers (SOX2 and nestin), and multipotency. Prominin-1-expressing cells from neonatal IC generated neurospheres, and culture of these neurospheres in differentiation-conditioned medium gave rise to gamma-aminobutyric acid-ergic (GABAergic) neurons, astrocytes, and oligodendrocytes. The presence of NSC-like cells in the IC has important implications for understanding IC development and for potential regenerative therapy.

**Electronic supplementary material:**

The online version of this article (doi:10.1007/s12035-017-0701-5) contains supplementary material, which is available to authorized users.

## Introduction

Almost all ascending projections from the lower auditory regions, including the cochlear nuclei, superior olivary complex, and lemniscus lateralis, converge in the inferior colliculus (IC) located on the roof of the midbrain. An ascending projection from IC projects into the medial geniculate body and subsequently to the auditory cortex [1]. Thus, the IC occupies a pivotal position for the integration of complex sound information. Indeed, damage to the bilateral IC by hemorrhage, infarction, or tumors induces severe hearing loss and disrupts speech recognition [2, 3]. In addition, 40% of the population older than 65 years has some degree of age-related hearing loss (presbycusis) [4] involving a combination of inner ear and central auditory system dysfunction [5]. Presbycusis with poor speech discrimination arises in part from the loss of the inhibitory neurotransmitter gamma-aminobutyric acid (GABA) in the IC [6, 7]. These reports suggest that therapeutic strategies targeting IC GABAergic neurons may be effective against presbycusis.

Recently, regenerative therapies using self-renewing multipotent stem cells have been developed and are expected to open new treatment possibilities for various diseases including inner ear and retro-cochlear hearing loss. Moreover, neural stem cells obtained from monkey ES cells were transplanted into the IC of rhesus monkeys, and these transplanted stem cells differentiated into mature neurons and responded to auditory stimuli [8]. Neural stem cells (NSCs) have been reported in auditory organs such as spiral ganglion, organ of Corti and stria vascularis [9], cochlea [10], and cochlear nucleus [11, 12]; however, it is unknown if NSCs also reside in the IC.

Prominin-1 (CD133) is a novel plasma membrane glycoprotein originally identified in mouse embryo neuroepithelium [13]. It was named “prominin” because of its notable localization in plasma membrane protrusions (from the Latin word “*prominere*,” meaning to be prominent) [13]. In humans, it is selectively expressed on hematopoietic stem or progenitor cells isolated from fetal liver, bone marrow, cord blood, and adult peripheral blood [14, 15]. Uchida et al. reported that Prominin-1^+^/CD34^−^/CD45^+^ cells from human fetal neural tissue have self-renewal potential and are capable for successful engraftment, migration, proliferation, and neural differentiation for prolonged periods following transplantation into the brain of NOD/SCID mice [16]. Moreover, Lee et al. demonstrated that Prominin-1^+^ lin^−^ (absence of markers of neuronal and glial lineages) cells from mouse postnatal cerebellum form neurospheres and differentiate into neurons and glial cells. Additionally, they also demonstrated that these cells generate neurons and glial cells upon transplant back into the cerebellum [17]. Prominin-1 is expressed in the fetal brain [16], postnatal cerebellum [17], adult subventricular zone [18], and hippocampus [19], suggesting utility as a marker for SCs in neural tissue as it is in the kidney [20], liver [21], pancreas [22], and skin [23]. However, SCs expressing prominin-1 have not been identified in the IC. Hence, we screened for prominin-1^+^ cells in the IC. Isolated prominin-1^+^ cells were then examined for NSC-like characteristics.

## Materials and Methods

### Animals

BALB/c mice were obtained from Shimizu Laboratory Supplies (Kyoto, Japan). Mice were housed at constant temperature and humidity within animal facilities under a 12-h light/dark cycle and provided food and water ad libitum. All experiments complied with protocols approved by the Kansai Medical University Animal Ethics Committee (12-054).

### Dissociation of the Inferior Colliculus

All mice were euthanized by cervical dislocation followed by decapitation. Inferior colliculi were dissected from mice at age < 1 week (neonatal IC), 1–2 weeks (1–2 w IC), and >3 weeks (post-weaning IC) as described previously [24]. Tissues were digested and dissociated to single cells using the Neural Tissue Dissociation Kit (P) or Neural Tissue Dissociation Kit (T) (Miltenyi Biotec, Bergisch Gladbach, Germany).

### Isolation and Culture of Prominin-1^+^ Cells

Prominin-1^+^ cells were selected from dissociated IC using a magnetic-activated cell sorting (MACS) system with anti-mouse prominin-1 magnetic beads (Miltenyi Biotec) according to the manufacturer’s protocol. To generate primary neurospheres, MACS-selected cells were cultured on uncoated plates at clonal density (1–2 cell/mm^2^) in Neurobasal medium (Life Technologies, CA, USA) containing MACS NeuroBrew-21 without vitamin A (Miltenyi Biotec) supplemented with epidermal growth factor (EGF) (20 ng/ml; Miltenyi Biotec), fibroblast growth factor-2 (FGF2) (20 ng/ml; Miltenyi Biotec), and penicillin-streptomycin-glutamine (Wako, Tokyo, Japan). The number of neurospheres was counted by microscopy following 6 days of culture at 37 °C under a 5% CO_2_ atmosphere. To generate secondary neurospheres, primary neurospheres were dissociated with Accutase (Life Technologies, Carlsbad CA, USA) and then seeded on new plates. For clonal cell analysis, prominin-1^+^ cells were seeded at one per well by the limiting dilution method or into 96-well plates (Sumitomo Bakelite, Tokyo, Japan) by a fluorescence-activated cell sorter (FACSAria I, BD). Clusters of cells > 50 μm in diameter were counted as neurospheres.

### Cell Differentiation From Neurospheres

To induce differentiation, neurospheres were replated on Matrigel (BD) thin-coated 8-well chamber slides (NUNC, Thermo Fisher Scientific, Waltham, MA, USA) with Neurobasal medium (Life Technologies) containing MACS NeuroBrew-21 (Miltenyi Biotec), 5% fetal bovine serum (Gibco, Life Technologies), leukemia inhibitory factor (LIF, 1 U/ml, ORF Genetics, Reykjavik, Iceland), all-trans retinoic acid (RA, 100 ng/ml, Sigma-Aldrich, St. Louis, MO, USA), and penicillin-streptomycin (Wako) for 7 days.

### Flow Cytometric Analysis

Cells prepared from neonatal or post-weaning IC were incubated with prominin-1-FITC, PSA-NCAM-APC, O4-APC, GLAST-Biotin, and A2B5-Biotin for 30 min at 4 °C following FcR blocking (2.4G2, BD Biosciences, Mississauga, Canada). Antibodies used are listed in Table S1. For visualization of immunostaining by biotin-conjugated primary antibodies, cells were stained with streptavidin (BD) conjugated to BV421 for 15 min at 4 °C. Dead cells were excluded by staining with propidium iodide. Stained cells were separated and counted using flow cytometry (FACS Aria I, BD) and FlowJo software (Tomy Digital Biology, Tokyo, Japan).

### Quantitative Real-Time PCR

Total RNA from IC tissue was extracted by TRIzol RNA Isolation Reagents (Life Technology) or ReliaPrep™ RNA Cell Miniprep System (Promega Corporation, Madison, WI, USA). Reverse transcription to the first-strand cDNA was performed using ReverTra Ace (TOYOBO, Osaka, Japan), and quantitative real-time PCR (qRT-PCR) was performed using SsoAdvanced™ Universal SYBR® Green Supermix (Bio-Rad, Hercules, CA, USA). Gene amplification was standardized to β-actin and gene expression level quantified as fold difference. Primer sequences are listed in Table S2.

### Immunohistochemistry and Immunofluorescence

To detect prominin-1, SOX2, and β-tubulin III (TUBB3) expressions in neonatal IC tissue, sections of the brain including the IC were fixed in 4% paraformaldehyde (PFA) overnight at 4 °C, embedded in O.C.T. Compound (Sakura Finetek, Tokyo, Japan), and dehydrated with 30% sucrose. Cryosections were sliced in the coronal plane at 20-μm thickness. For antigen retrieval, sections were activated in Histo VT One solution (Nacalai Tesque, Kyoto, Japan) for 20 min at 70 °C. Sections were then incubated with a blocking solution of phosphate-buffered saline (PBS) containing 2% Block Ace (DS Pharma Biomedical, Osaka, Japan) and 0.3% Triton X-100 (Sigma-Aldrich) for 45 min at room temperature (RT). Blocked sections were stained with antibodies against prominin-1, SOX2, or β-tubulin III overnight at 4 °C. After washing in PBS-0.1% BSA, sections were incubated with an appropriate secondary antibody for 1 h at RT. Sections were counterstained with 4,6-diamidino-2-phenylindole (DAPI) (Dojindo, Kumamoto, Japan) and mounted using Fluorescence Mounting Medium (Dako, Carpinteria, CA, USA). Images were analyzed by LSM 700 (Carl Zeiss, Jena, Germany).

### Statistical Analysis

Data are presented as mean ± SEM. Group differences were evaluated for statistical significance using the Mann-Whitney test. The threshold of significance was set at *p* < 0.05 for all tests. All statistical analyses were performed using Statcel-The Useful Add-in Forms on Excel-3rd ed.

## Results

### Neonatal IC Expresses Immature Cell Markers

**Fig. 1:**
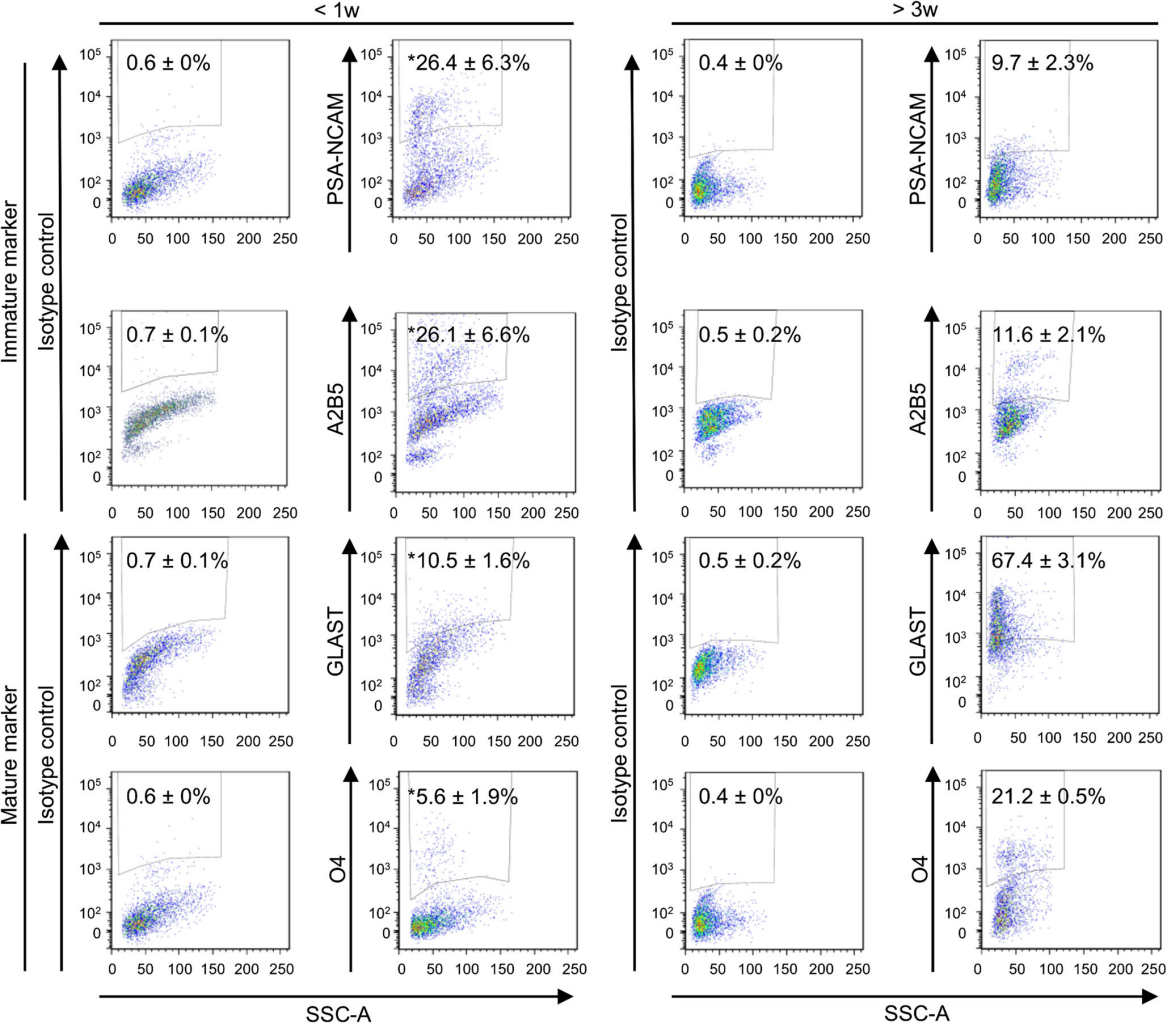
Surface expression of immature and mature markers on inferior colliculus (IC) cells. Surface marker expression was analyzed by flow cytometry. *Left* and *right panels* are cell counts from neonatal IC (< 1 week) and post-weaning IC (> 3 weeks), respectively (*n* = 3 for both). *Upper* and *lower panels* are counts of cells expressing immature (PSA-NCAM and A2B5) and mature (GLAST and O4) markers, respectively. Numbers are relative proportions (mean ± SEM). *Statistically different (*p* < 0.05) from post-weaning IC. *SSC-A*, side scatter gating

Surface markers on IC cells were analyzed by flow cytometry. As shown in Fig. 1, large subpopulations of cells dissociated from neonatal IC expressed the immature neuron marker PSA-NCAM (26.4 ± 6.3%) or the glial precursor cell marker A2B5 (26.1 ± 6.6%), while expression of these markers was significantly lower in cells from post-weaning IC (PSA-NCAM 9.7% ± 2.3%, A2B5 11.6% ± 2.1%; both *p* < 0.05). In contrast, fewer neonatal IC cells expressed the mature astrocyte marker GLAST (10.5 ± 1.6%) or the oligodendrocyte marker O4 (5.6 ± 1.9%) than cells isolated from post-weaning IC (GLAST 67.4% ± 3.1%, O4 21.2 ± 0.5%; both *p* < 0.05). These data suggest that stem cells do exist in IC.

### Prominin-1 Is Highly Expressed in Neonatal IC

**Fig. 2:**
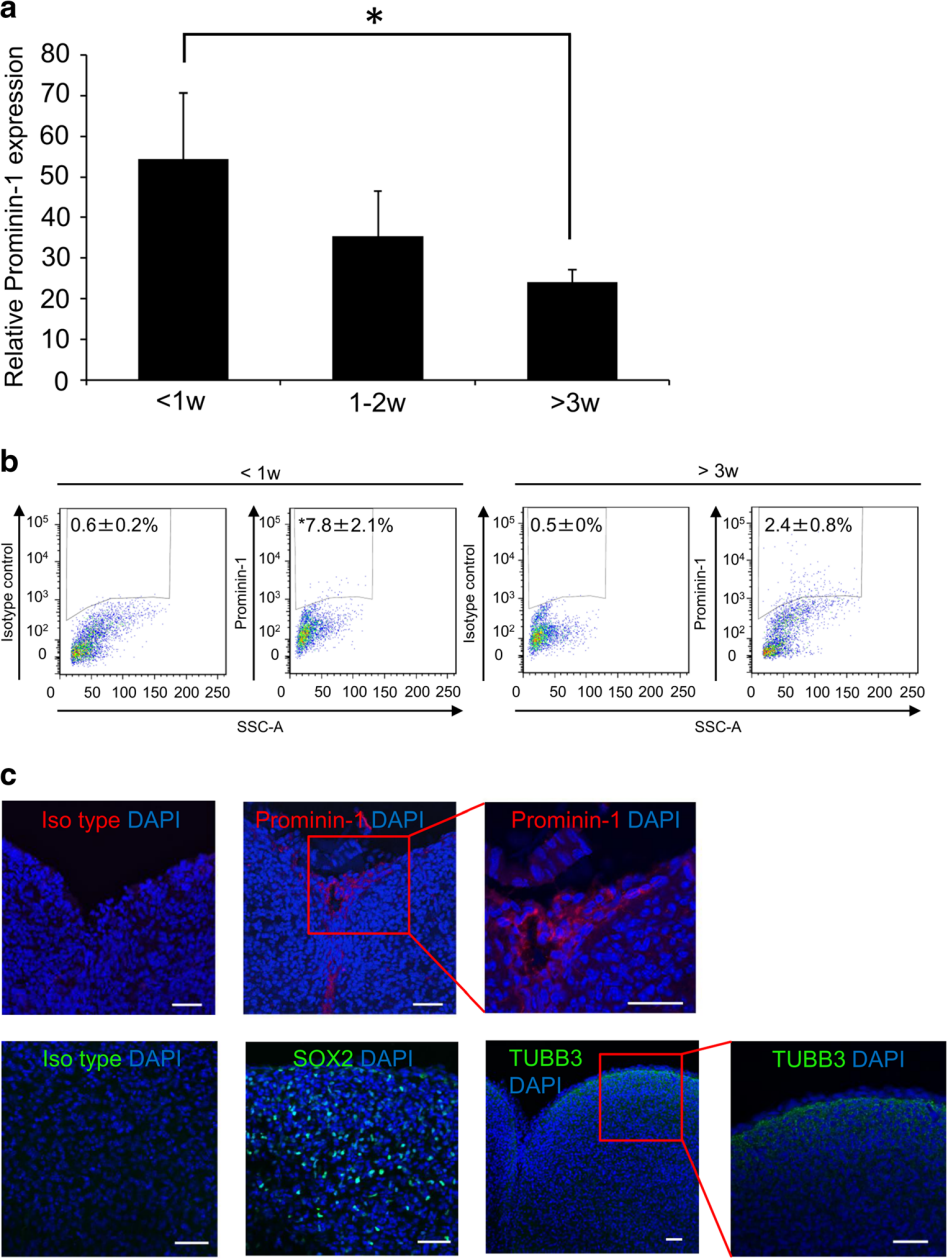
Expression of prominin-1 in neonatal IC. **a** The quantitative real-time PCR analysis of prominin-1 expression in neonatal IC (< 1 week), 1–2-week-old IC (1–2 weeks), post-weaning IC (> 3 weeks), and prominin-1-negative neonatal IC cells. Relative gene expression levels were calculated by the 2^−ΔΔCT^ method with prominin-1 negative neonatal IC cells as the reference. Data are expressed as mean ± SEM (< 1 week, *n* = 12; 1–2 weeks, *n* = 10; > 3 weeks, *n* = 9; prominin-1-negative neonatal IC cells, *n* = 3). **b** Flow cytometric analysis of prominin-1 expression by mouse neonatal (*left panels*) and post-weaning IC (*right panels*) cells following cell dissociation. **c** Immunofluorescence images of fixed IC sections stained with antibodies against prominin-1, SOX2, and TUBB3, and corresponding isotype controls. *Scale bar*: 50 μm. *Statistically different (*p* < 0.05) from post-weaning IC

Prominin-1 is also considered an NSC marker [16–19], so we investigated prominin-1 expression in neonatal IC, 1–2 w IC, and post-weaning IC cells by qRT-PCR, flow cytometry, and immunohistochemistry. Prominin-1 messenger RNA (mRNA) expression in neonatal IC was significantly higher than in post-weaning IC (*p* < 0.05), while there was no significant difference between neonatal IC and 1–2w IC (Fig. 2a). Likewise, flow cytometry (Fig. 2b) revealed that prominin-1 was expressed on more neonatal IC cells (7.8 ± 2.1%), than post-weaning IC cells (2.4 ± 0.8%) (*p* < 0.05). Neonatal IC cells from dorsal to lateral regions expressed prominin-1 at the surface (Fig. 2c, Fig. S1a). In addition, neonatal IC cells in situ expressed the NSC marker SOX2 (Fig. 2c). Additionally, TUBB3, a mature neuron marker, is mainly expressed on the surface under the prominin-1-expressed layer of neonatal IC, whereas SOX is diffusely expressed (Fig. 2c). Taken together, prominin-1 appears to be highly expressed in the neonatal IC at both the mRNA and protein levels.

### Prominin-1^+^ Cells Isolated From Neonatal IC Generate Neurospheres

**Fig. 3:**
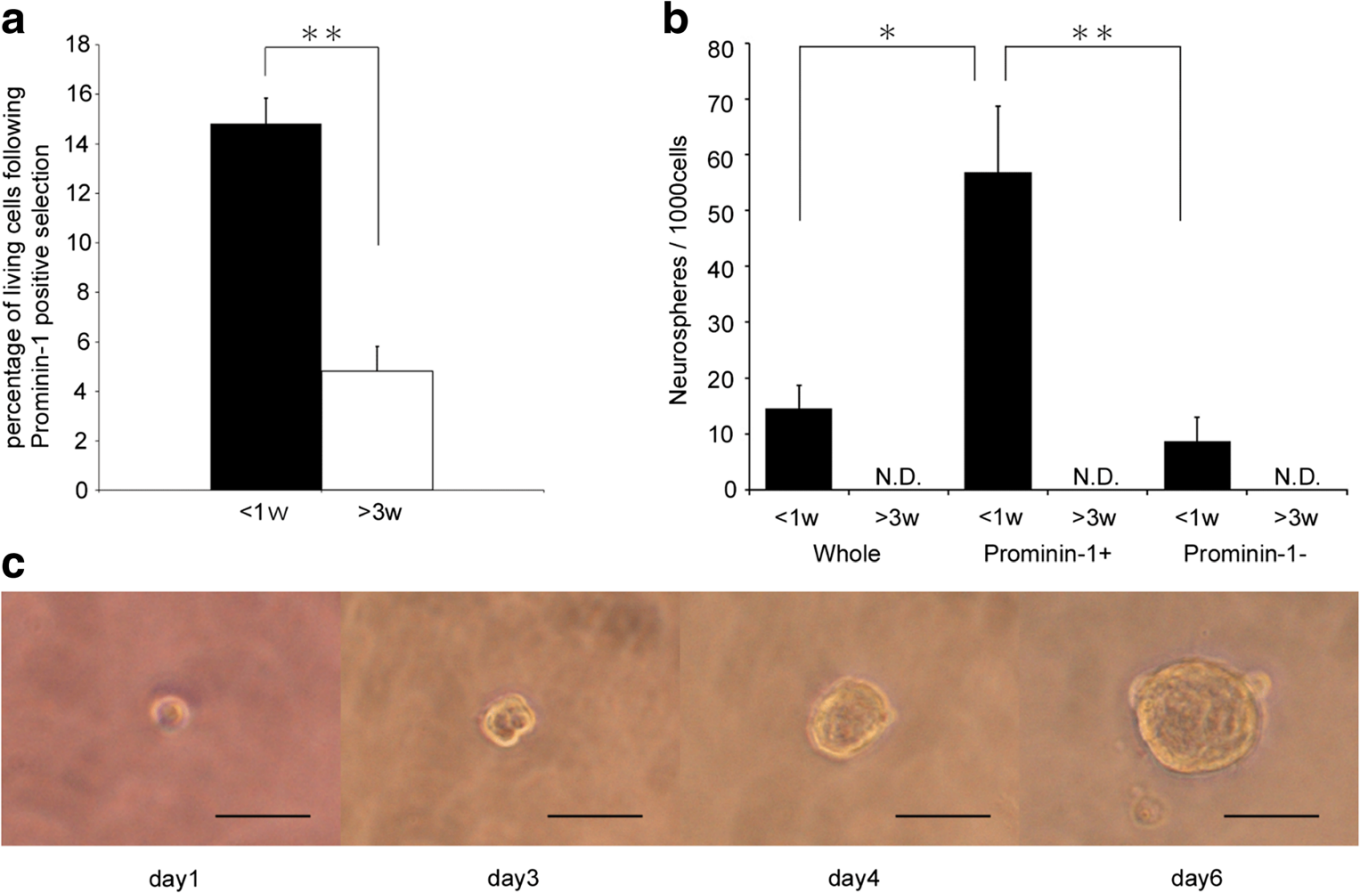
Generation of neurospheres from prominin-1^+^ neonatal IC cells. **a** Recovery rates of viable prominin-1^+^ cells by MACS from neonatal (> 1 week) and post-weaning (> 3 weeks) IC. Data are expressed as mean ± SEM (*n* = 8). **b** Numbers of neurospheres generated per 1000 cells from unseparated (whole) or separated cells by prominin-1^+^ selection (indicated as prominin-1^+^ and prominin-1^−^) in neonatal (*black bar*) and post-weaning IC (*opened ba*r). Data are expressed as mean ± SEM (*n* = 8). **c** Time lapse analysis from day 1 until day 6 in culture for a single prominin-1^+^ cell from neonatal IC. *Scale bar*: 50 μm. Statistically different from post-weaning IC or prominin-1-positive cells: * *p* < 0.05 and ** *p* < 0.01

Prominin-1 was highly expressed in neonatal IC compared to post-weaning IC, suggesting an association with immature cell status. We thus investigated whether prominin-1^+^ cells from neonatal IC have SC properties. We first examined if these cells have self-renewal capacity by examining if neurospheres are formed when these cells are seeded at clonal density. Prominin-1^+^ cells were isolated from the dissociated IC population using a MACS system. The proportion of surviving prominin-1^+^ cells was significantly higher from neonatal IC than post-weaning IC (*p* < 0.01) (Fig. 3a), consistent with qRT-PCR and flow cytometry. Selected prominin-1^+^ cells from neonatal IC plated in serum-free medium containing EGF and FGF2 at 1–2 cells/mm^2^ (clonal density) generated significantly more neurospheres than the whole cell population (*p* < 0.05) or the prominin-1^−^ population (*p* < 0.01) (Fig. 3b). By contrast, no neurospheres were generated from post-weaning IC cells, even those expressing prominin-1. Furthermore, we found that single prominin-1^+^ cells isolated from neonatal IC generated individual neurospheres (Fig. 3c) in the limiting dilution assay [25]. Moreover, primary neurospheres generated secondary neurospheres with similar morphology after dissociation (11.3 ± 7 neurospheres/1000 cells) (Fig. S2). Thus, at least a fraction of prominin-1^+^ cells isolated from the neonatal IC have a proliferative and self-renewal capacity, and cardinal features of NSCs.

### Prominin-1^+^ Cells Express Other Known Neural Stem Cell Markers

**Fig. 4:**
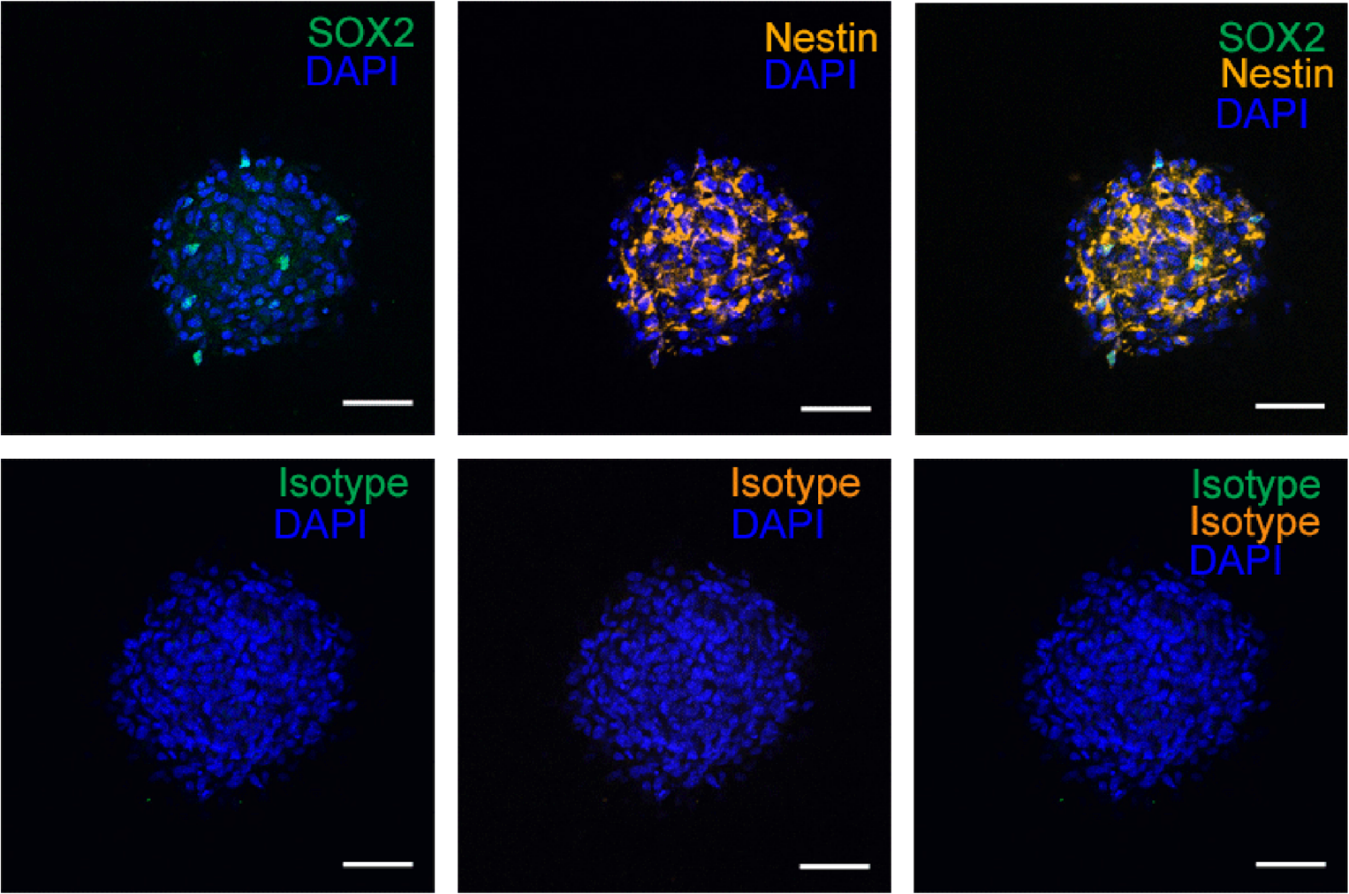
Expression of immature markers on cells from prominin-1^+^ cell-generated neurospheres. Immunofluorescence staining with SOX2 and nestin in prominin-1^+^ cell-generated neurospheres from neonatal IC. Nuclei were counterstained with DAPI. Corresponding isotypes were used as negative controls. *Scale bar*: 50 μm

To further characterize the stemness of prominin-1^+^ cells, we performed immunocytochemical staining for other known NSC markers (Fig. S3). Spheres generated from prominin-1^+^ cells isolated from neonatal IC also expressed the NSC markers SOX2 [26] and nestin (Fig. 4), similar to multipotent cells in neurospheres isolated from the cerebellum [17], subventricular zone [18, 27], and hippocampus [19].

### Neurospheres Generated From Prominin-1^+^Cells Have the Potential to Differentiate into Neurons and Glia

**Fig. 5:**
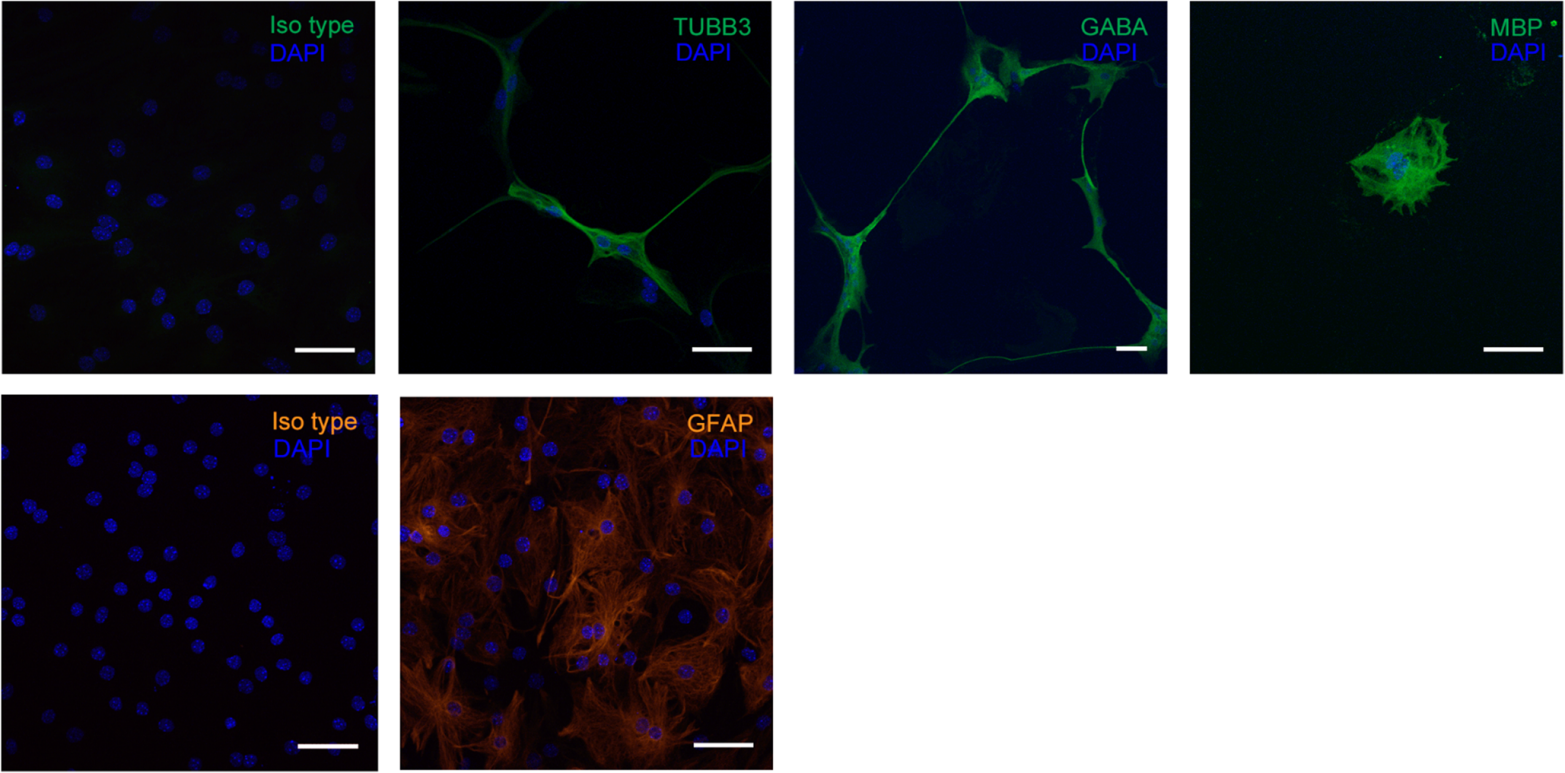
Cells within prominin-1^+^ cell-generated neurospheres differentiate into neurons and glia. Following induction in differentiation medium, immunofluorescence staining with mature neural markers β-tubulin III (TUBB3), myelin basic protein (MBP), and glial fibrillary acid protein (GFAP) identified neurons, oligodendrocytes, and astrocytes, respectively. GABA was also expressed in a subpopulation of neurons. Corresponding isotypes were used as negative controls. *Scale bar*: 50 μm

To investigate whether prominin-1^+^ cells in neonatal IC neurospheres do exhibit multipotent differentiation, neurospheres were cultured in conditioned differentiation medium and mature phenotypes identified by immunohistochemical staining for the neuronal marker β-tubulin III (and GABA), the astrocytic marker GFAP, and the oligodendrocyte marker MBP. Indeed, neurospheres generated from neonatal IC prominin-1^+^ cells gave rise to GABAergic neurons, astrocytes, and oligodendrocytes (Fig. 5).

## Discussion

Neural stem cells have been identified in several brain regions but not in the IC, a critical region of the central auditory pathway showing frequent age-related dysfunction [4–7]. Here, we show that neonatal IC possesses cells with the cardinal NSC properties: self-renewal capacity and multipotency. First, significantly larger subpopulations of cells in neonatal IC expressed immature neural cell markers compared to post-weaning IC, including PSA-NCAM, A2B5, and the surface protein prominin-1. Second, prominin-1^+^ cells isolated from neonatal IC by MACS expressed the known stem cell markers SOX2 and nestin. Third, prominin-1^+^ cells from neonatal IC formed neurospheres when cultured at clonal density in serum-free medium containing EGF and FGF2, and dissociated cells from these neurospheres formed secondary neurospheres, confirming self-renewal capacity. Finally, neurospheres cultured in differentiation medium formed cells expressing markers for mature GABAergic neurons, astrocytes, and oligodendrocytes. The presence of stem-like cells in neonatal IC suggests a potential new regenerative therapy for auditory dysfunction involving IC degeneration or lesions.

Selection of appropriate enzymes is critical for efficient isolation of functional cells from tissue. One previous study isolated prominin-1^+^ cells from human neural tissues and other tissues using trypsin for dissociation before FACS [16]. Therefore, we also dissociated neonatal mouse IC with trypsin (Neural Tissue Dissociation Kit T), but this resulted in low neurosphere yield. By contrast, another study reported that papain (Neural Tissue Dissociation Kit P) effectively dissociated postnatal rat cerebellum [17]. Further, a study describing dissociated culture of the neonatal (postnatal day 3–5) rat IC reported low neuronal yield and glial cells with few branched processes unless papain was used for dissociation [24]. Similarly, we found tenfold higher neurosphere yield using papain for IC dissociation compared to trypsin (data not shown). Thus, trypsin appears to exert higher toxicity upon stem-like cells in neonatal mouse IC than papain.

Prominin-1 expression in the mouse hippocampus was found to be relatively stable with age [19]. While we also found expression of prominin-1 in both developing and post-weaning mouse IC, expression was significantly higher in neonatal IC. In accordance with our results, prominin-1 was expressed on 16.5 ± 4.6% of cells from the forebrain germinal zone of the murine brain at embryonic day 12.5, but by only 3.2 ± 1.4% of adult forebrain subventricular cells [18]. Moreover, we found that prominin-1^+^ cells produced more neurospheres than prominin-1^−^ cells from the postnatal IC, in accordance with previous reports of neurosphere formation from prominin-1^+^ cells in the postnatal cerebellum [17], forebrain [18], and hippocampus [19]. Additionally, single prominin-1^+^ cells from neonatal IC formed neurospheres at clonal density (data not shown).

A previous study reported that some neurons differentiated from cerebellar prominin-1^+^ cell-derived neurospheres expressed GABA, the main inhibitory neurotransmitter in the central auditory system [17]. Decreased GABA expression by IC neurons is associated with presbycusis in animal models [6, 7], while the total number of IC neurons is independent of age [28]. In humans, presbycusis is characterized by a dramatic loss of speech understanding without a parallel change in pure-tone threshold and decreased abilities to localize sound and comprehend speech in noise. Reduced GABA levels were found in Heschl’s gyri (primary auditory cortex) of patients with presbycusis [29]. Further, expression of glutamate decarboxylase (GAD), the key enzyme for GABA synthesis, was also decreased with age in IC neurons [30].

These observations strongly suggest that a reduction in GABA within the IC contributes to age-related central presbycusis, and we found that prominin-1^+^ cells from IC could be induced to differentiate into GABAergic neurons.

In this study, we conclude that neural stem cells expressing prominin-1 exist in IC, particularly in neonatal mice, and differentiate into GABAergic neurons. Interestingly, Revoltella et al. reported that transplantation of human cord blood prominin-1^+^ hematopoietic stem cells into the inner ear of oto-injured mice improved cochlear function [31]. Therefore, the development of an efficient method to analyze the differentiation of neural stem cells expressing prominin-1 into GABAergic neurons may be a new strategy to cure presbycusis using regenerative medicine.

## Electronic Supplementary Material

Table S1 (DOCX 19 kb)

Table S2 (DOCX 16 kb)

Fig. S1 Prominin-1 in neonatal IC. Immunofluorescence (IF) staining for prominin-1 in neonatal IC. IF images show the surface of IC tissue. Scale bar: 100 μm (GIF 1218 kb)

High resolution image (TIFF 1152 kb)

Fig. S2 Transgeneration of neurospheres. Microscope image of a secondary neurosphere generated from a primary neurosphere. Primary and secondary neurospheres show similar gross morphology. Scale bar: 50 μm (GIF 591 kb)

High resolution image (TIFF 248 kb)

Fig. S3 Expression of immature markers on Prominin-1^+^ cells. Immunofluorescence staining for SOX2 in prominin-1^+^ cells isolated from neonatal IC. Nuclei were counterstained with DAPI. The corresponding isotype was used as a negative control. Scale bar: 50 μm (GIF 191 kb)

High resolution image (TIFF 87 kb)
