# Supplementary material for: Cells Expressing Prominin-1 in Neonatal Murine Inferior Colliculus Differentiate into Neurons and Glia
Source: Mol Neurobiol. 2017 Aug 9;55(6):4998–5005. doi: 10.1007/s12035-017-0701-5 (PMC5948249; doi:10.1007/s12035-017-0701-5)
Supplement: Supplementary file 1 — (DOCX 19 kb) [file 12035_2017_701_MOESM1_ESM.docx]

**Online Supplementary Tables**

**Table S1. List of antibodies**

|  | Clone | Supplier | Dilution | Isotype |
| --- | --- | --- | --- | --- |
| 1^st^ Antibody |  |  |  |  |
| rat anti mouse CD133 (Prominin-1) | 13A4 | eBioscience | 1:50 | rat IgG1 |
| rat anti mouse CD133 (Prominin-1) FITC | 13A4 | eBioscience | 1:50 | rat IgG1 |
| rat anti mouse SOX2 Alexa Fluor® 488 | Btjce | eBioscience | 1:50 | rat IgG2a-488 |
| mouse anti mouse PSA-NCAM APC | 2-2B | Miltenyi Biotec | 1:20 | mouse IgM |
| mouse anti mouse Nestin | Rat-401 | eBioscience | 1:50 | mouse IgG1 |
| mouse anti mouse A2B5 Biotin | 105HB29 | Miltenyi Biotec | 1:20 | mouse IgM |
| rabbit anti mouse Myelin Basic Protein (MBP) | polyclonal | Sigma | 1:100 | rabbit IgG |
| mouse anti mouse O4 APC | O4 | Miltenyi Biotec | 1:20 | mouse IgM |
| rabbit anti mouse β-Tubulin III | polyclonal | Sigma | 1:50 | rabbit IgG |
| mouse anti mouse GFAP | GA5 | CST | 1:300 | mouse IgG1 |
| mouse anti mouse GLAST Biotin | ACSA-1 | Miltenyi Biotec | 1:20 | mouse IgG2a |
| rabbit anti mouse GABA | polyclonal | Sigma | 1:100 | rabbit IgG |
| 2^nd^ Antibody |  |  |  |  |
| conjugated with CF™ 555A Goat Anti-rat IgG |  | Biotium | 1:200 |  |
| conjugated with CF™ 488A Goat Anti-rabbit IgG |  | Biotium | 1:200 |  |
| conjugated with CF™ 647A Goat Anti-mouse IgG |  | Biotium | 1:200 |  |
| conjugated with BD Horizon™ BV421 Streptavidin |  | BD | 1:200 |  |
