# Supplementary material for: Cells Expressing Prominin-1 in Neonatal Murine Inferior Colliculus Differentiate into Neurons and Glia
Source: Mol Neurobiol. 2017 Aug 9;55(6):4998–5005. doi: 10.1007/s12035-017-0701-5 (PMC5948249; doi:10.1007/s12035-017-0701-5)
Supplement: Supplementary file 2 — (DOCX 16 kb) [file 12035_2017_701_MOESM2_ESM.docx]

**Table S2. List of primers**

|  | **Forward** | **Reverse** |
| --- | --- | --- |
| **β-actin** | 5’-AAATCGTGCGTGACATCAAAG-3’ | 5’-AAGAAGGAAGGCTGGAAAAGAG-3’ |
| **Prominin-1** | 5’-TGAAGAAGATCCTTGCCTC-3’ | 5’-TCCGCAACATAGCCACAC-3’ |
