# Supplementary figures and images for: Cells Expressing Prominin-1 in Neonatal Murine Inferior Colliculus Differentiate into Neurons and Glia
Source: Mol Neurobiol. 2017 Aug 9;55(6):4998–5005. doi: 10.1007/s12035-017-0701-5 (PMC5948249; doi:10.1007/s12035-017-0701-5)

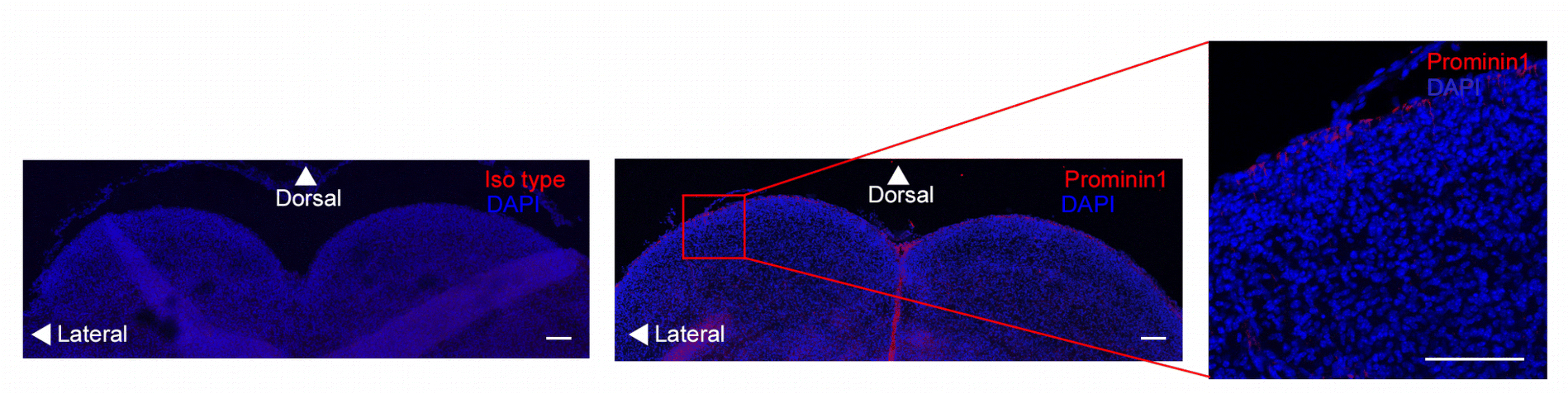

Supplement: Supplementary file 3 — Prominin-1 in neonatal IC. Immunofluorescence (IF) staining for prominin-1 in neonatal IC. IF images show the surface of IC tissue. Scale bar: 100 μm (GIF 1218 kb) [file 12035_2017_701_Fig6_ESM.gif]

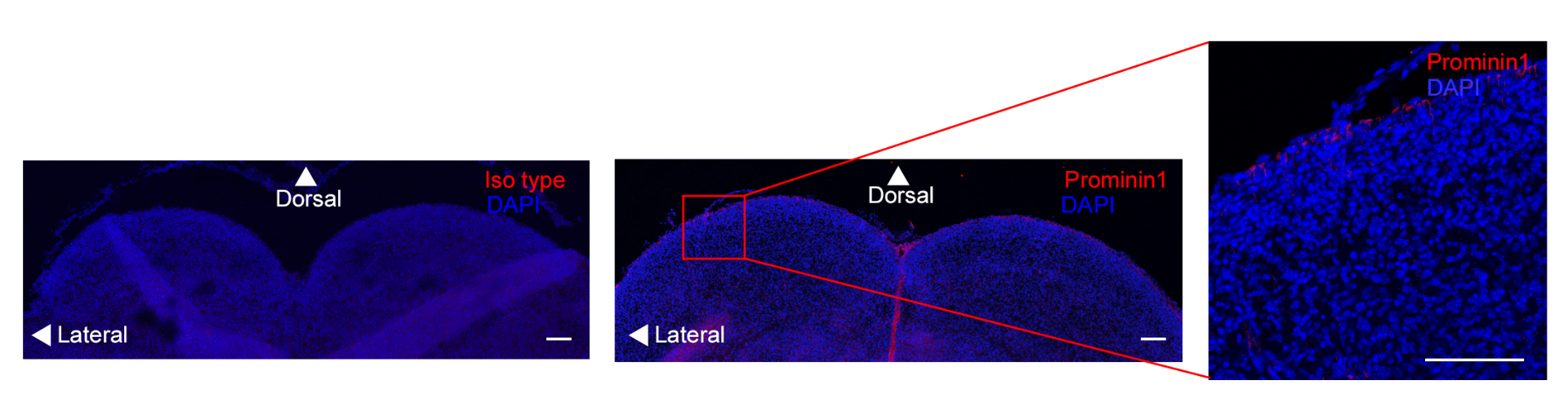

Supplement: Supplementary file 4 — High resolution image (TIFF 1152 kb) [file 12035_2017_701_MOESM3_ESM.tif]

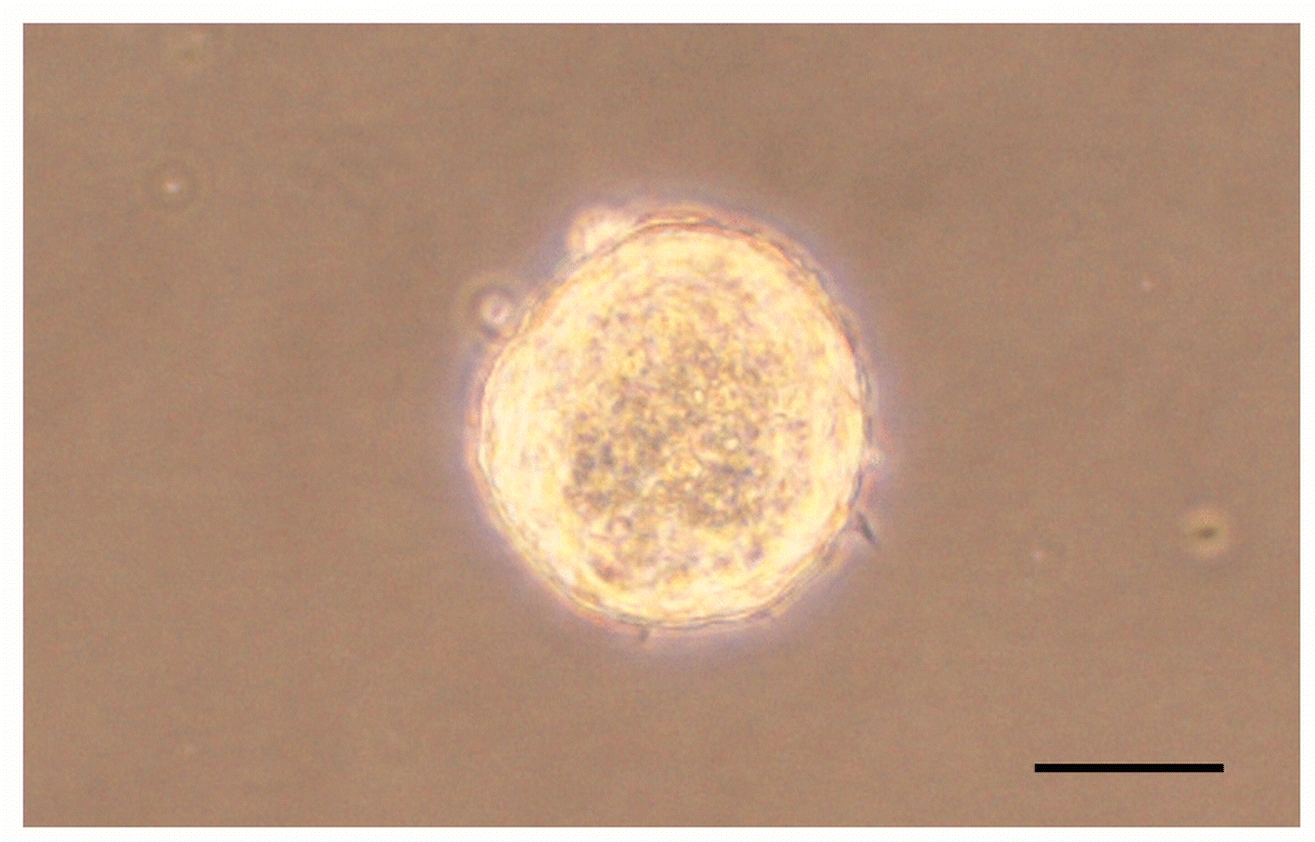

Supplement: Supplementary file 5 — Transgeneration of neurospheres. Microscope image of a secondary neurosphere generated from a primary neurosphere. Primary and secondary neurospheres show similar gross morphology. Scale bar: 50 μm (GIF 591 kb) [file 12035_2017_701_Fig7_ESM.gif]

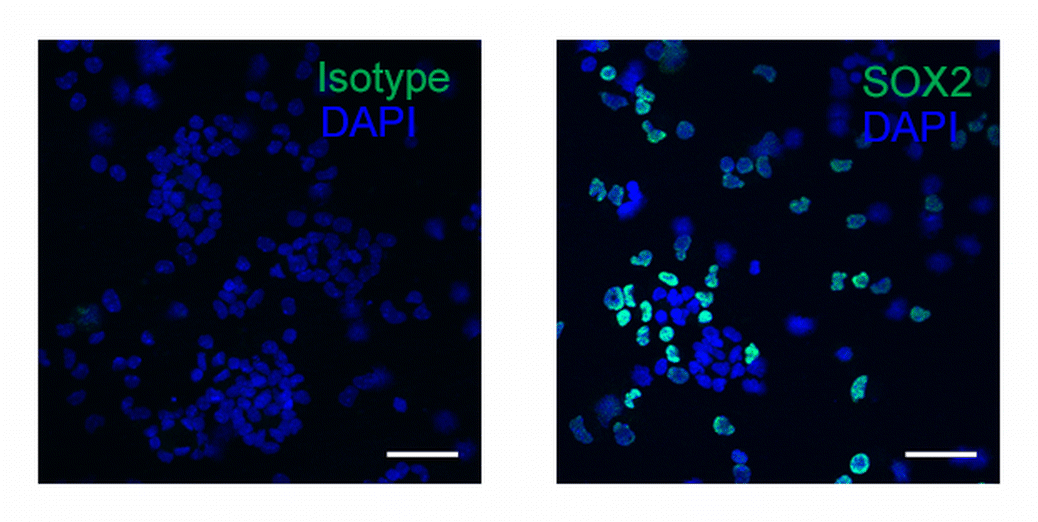

Supplement: Supplementary file 7 — Expression of immature markers on Prominin-1+ cells. Immunofluorescence staining for SOX2 in prominin-1+ cells isolated from neonatal IC. Nuclei were counterstained with DAPI. The corresponding isotype was used as a negative control. Scale bar: 50 μm (GIF 191 kb) [file 12035_2017_701_Fig8_ESM.gif]
